# Supplementary figures and images for: Integrated Multi-Omic Analysis Reveals Novel Subtype-Specific Regulatory Interactions in Pediatric B-Cell Acute Lymphoblastic Leukemia
Source: Cancers (Basel). 2026 Mar 3;18(5):813. doi: 10.3390/cancers18050813 (PMC12984152; doi:10.3390/cancers18050813)

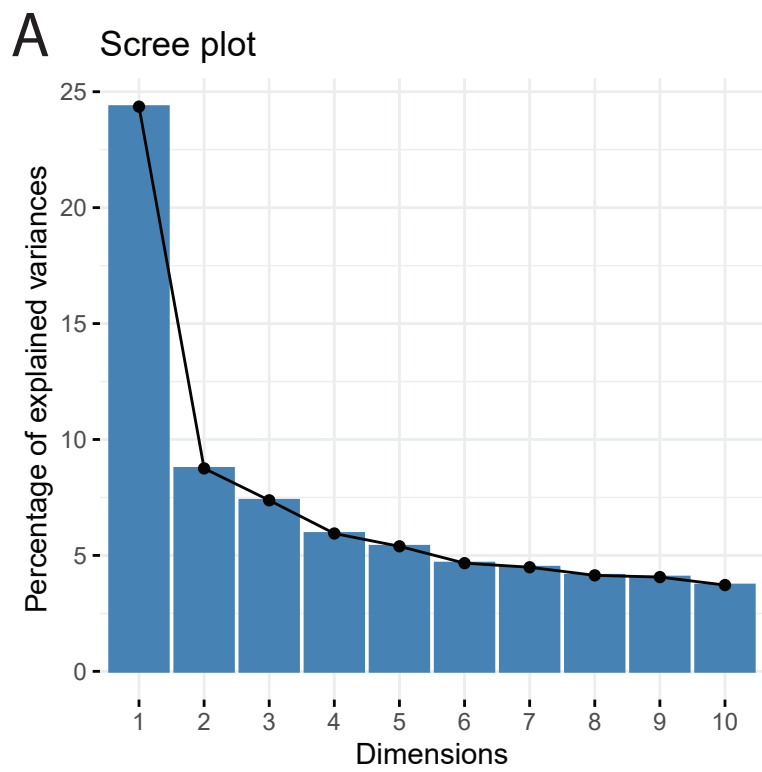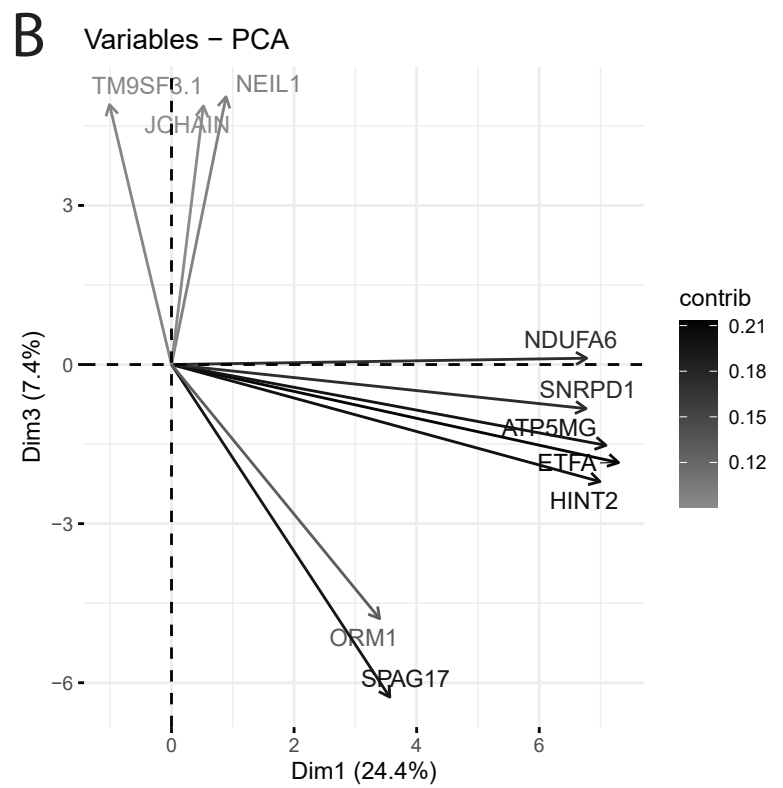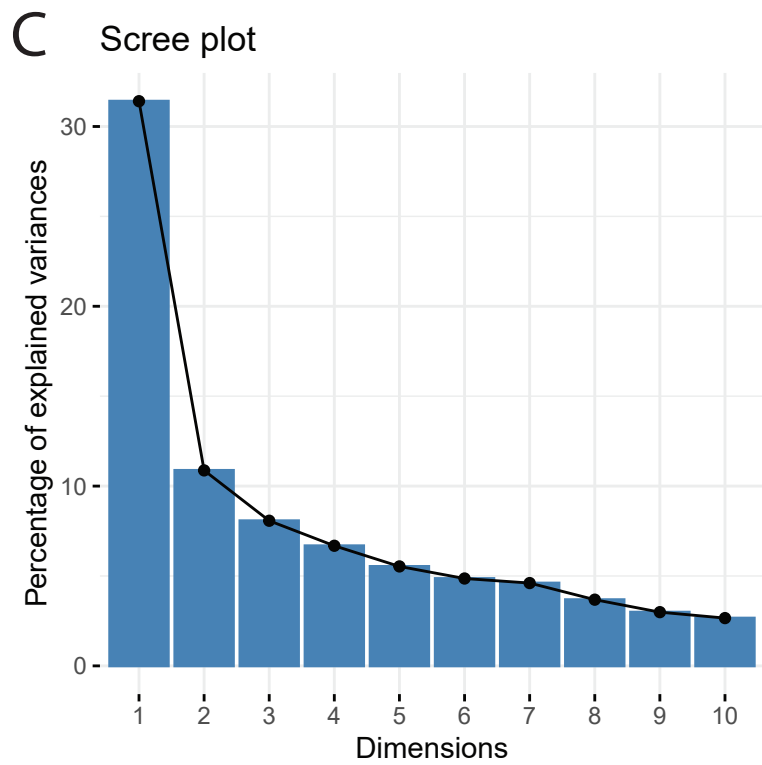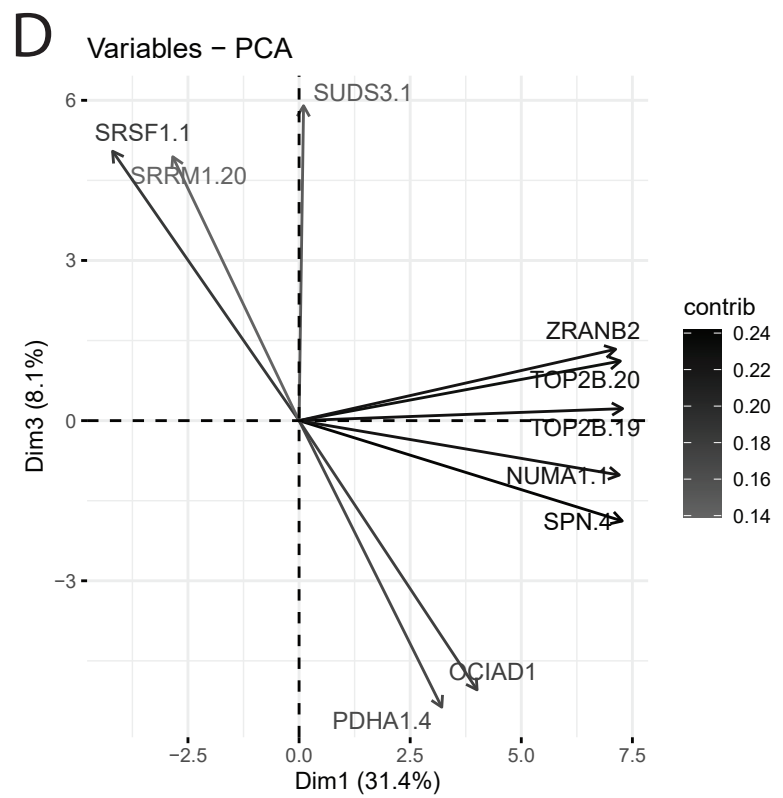

Supplement: Supplementary file 1 [file cancers-18-00813-s001.zip › SupplementaryFigure1.pdf]
